# Cardiac Reverse Remodeling in Ischemic Heart Disease with Novel Therapies for Heart Failure with Reduced Ejection Fraction

**DOI:** 10.3390/life13041000

**Published:** 2023-04-13

**Authors:** Sabina Andreea Leancă, Irina Afrăsânie, Daniela Crișu, Iulian Theodor Matei, Ștefania Teodora Duca, Alexandru Dan Costache, Viviana Onofrei, Ionuţ Tudorancea, Ovidiu Mitu, Minerva Codruța Bădescu, Lăcrămioara Ionela Șerban, Irina Iuliana Costache

**Affiliations:** 1 Cardiology Clinic, “St. Spiridon” County Clinical Emergency Hospital, 700111 Iași, Romania; 2 Department of Internal Medicine, “Grigore T. Popa” University of Medicine and Pharmacy, 700115 Iași, Romania; 3 Department of Cardiovascular Rehabilitation, Clinical Rehabilitation Hospital, 700661 Iași, Romania; 4 Department of Physiology, “Grigore T. Popa” University of Medicine and Pharmacy, 700115 Iași, Romania; 5 Internal Medicine Clinic, “St. Spiridon” County Clinical Emergency Hospital, 700111 Iași, Romania

**Keywords:** heart failure, ischemic heart disease, myocardial infarction, left ventricular remodeling

## Abstract

Despite the improvements in the treatment of coronary artery disease (CAD) and acute myocardial infarction (MI) over the past 20 years, ischemic heart disease (IHD) continues to be the most common cause of heart failure (HF). In clinical trials, over 70% of patients diagnosed with HF had IHD as the underlying cause. Furthermore, IHD predicts a worse outcome for patients with HF, leading to a substantial increase in late morbidity, mortality, and healthcare costs. In recent years, new pharmacological therapies have emerged for the treatment of HF, such as sodium-glucose cotransporter-2 inhibitors, angiotensin receptor-neprilysin inhibitors, selective cardiac myosin activators, and oral soluble guanylate cyclase stimulators, demonstrating clear or potential benefits in patients with HF with reduced ejection fraction. Interventional strategies such as cardiac resynchronization therapy, cardiac contractility modulation, or baroreflex activation therapy might provide additional therapeutic benefits by improving symptoms and promoting reverse remodeling. Furthermore, cardiac regenerative therapies such as stem cell transplantation could become a new therapeutic resource in the management of HF. By analyzing the existing data from the literature, this review aims to evaluate the impact of new HF therapies in patients with IHD in order to gain further insight into the best form of therapeutic management for this large proportion of HF patients.

## 1. Introduction

Heart failure (HF) is a clinical syndrome described as the incapacity of the heart to maintain the necessary blood flow in order to satisfy the body’s requirements. It is caused by structural and functional anomalies of the myocardium which result in impaired ventricular filling or blood ejection [1,2]. Although a broad spectrum of diseases such as hypertension, diabetes, or valvular diseases are potential causes of HF, ischemic heart disease (IHD) remains the main pathology leading to HF, and it portends a worse outcome, leading to a substantial increase in late morbidity, mortality, and healthcare costs [3,4]. 

The process involved in HF progression is currently known as cardiac remodeling and it includes a series of molecular, cellular, and interstitial changes which result in alterations in left ventricular (LV) size, shape, and function [1,5,6]. In IHD, the LV ejection fraction (EF) is frequently reduced, and the LV undergoes eccentric remodeling with normal or decreased wall thickness, and increased end-systolic and end-diastolic volumes [7,8,9]. 

Novel therapies including revolutionary drugs such as angiotensin receptor-neprilysin inhibitors (ARNI) and sodium-glucose co-transporter-2 (SGLT2) inhibitors have demonstrated their effectiveness in improving outcomes and promoting reverse remodeling in HF with reduced EF (HFrEF). Although coronary revascularization therapy limits the progression to cardiac dysfunction and adverse LV remodeling, a significant percentage of patients with IHD continue to develop HFrEF [10,11]. 

In the first part of this narrative review, we have addressed the main pathophysiological mechanisms that promote ventricular remodeling in IHD, which could represent potential therapeutic targets to counteract cardiac maladaptive changes. In the second part, we have discussed the results of recent HFrEF clinical trials that have employed novel therapeutic pharmacological agents and devices, with the purpose of exploring existing knowledge on their potential role in IHD. 

## 2. Ventricular Remodeling in IHD

In acute myocardial infarction (MI), the process of LV remodeling begins rapidly, within the first hours of ischemia, as myocyte loss will initiate a cascade of immuno-inflammatory pathways and cellular activities such as complement activation, the production of reactive oxygen species (ROS) and the activation of inflammasomes. The repair process begins with the action of inflammatory cells which will replace the necrotic myocardium with granulation tissue, while fibroblasts will form a new collagen matrix that contributes to the formation of the post-infarction scar in the infarcted area [12,13,14]. This phase is essential, as it preserves the structural integrity of the infarcted area and it prevents the rupture of the ventricular wall, but it also evolves along with the thinning and elongation of the myocardial fibers due to the increased stress wall in conditions of overload. The activation of the sympathetic nervous system (SNS) and renin-angiotensin-aldosterone system (RAAS) occurs initially as a compensatory mechanism in the acute decompensated state to become later a maladaptive process which will sustain the remodeling processes. These mechanisms will alter the LV configuration, from an initial elliptical shape to a spherical, dilated LV, with increased end-systolic and end-diastolic volumes and reduced cardiac output [15,16]. 

**Figure 1:**
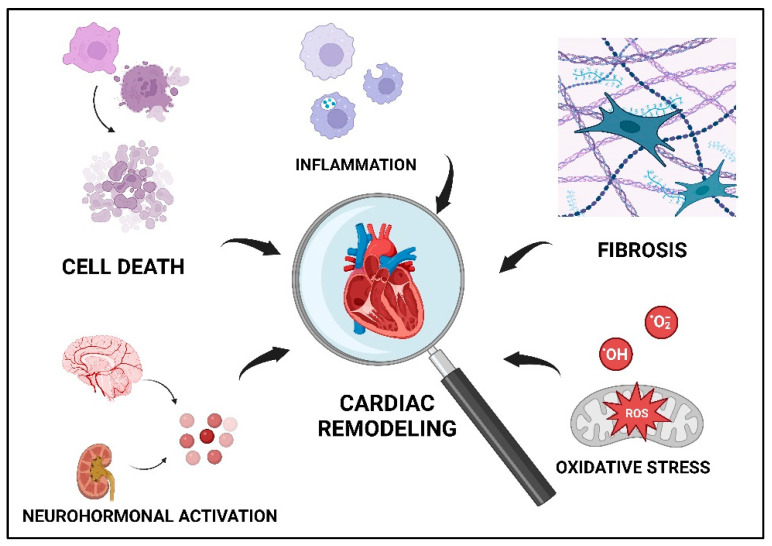
Mechanisms involved in the appearance and progression of cardiac remodeling in IHD. ROS, reactive oxygen species.

Several mechanisms involved in the appearance and progression of cardiac remodeling are discussed below and summarized in Figure 1. 

### 2.1. Cell Death

Myocyte loss is a key factor in the development of ventricular remodeling in IHD. During ischemic conditions, cell death may be induced by activating an intrinsic pathway originating in the mitochondria and an extrinsic pathway mediated by several cell death receptors such as tumor necrosis factor (TNF) receptors [17]. As a result, processes such as apoptosis and necrosis are activated and lead to impaired contractility which will trigger the remodeling of the myocardium and ultimately progression to HF [18,19,20]. 

During the initial phase of MI, the more predominant process is apoptosis, a form of programmed, non-lytic cell death, which consists of cell shrinkage, nuclear fragmentation, and chromatin condensation [21,22,23]. As the apoptotic bodies are cleared by phagocytes, the process does not evolve with subsequent inflammatory response [24]. In the healing phases, apoptosis can predict the severity of myocardial remodeling as increased apoptotic activity is correlated with higher degrees of remodeling [25]. In contrast, necrosis is characterized by the disintegration of the plasma membrane followed by the swelling of cells and their organelles, including mitochondria, and the collapse of intracellular homeostasis. Due to the loss of membrane integrity, the cellular content is released, triggering an intense inflammatory reaction that contributes to myocardial repair but also to adverse ventricular remodeling [25,26,27,28]. Several studies recognized the existence of a different type of programmed cell death commonly present in MI, which shares similarities with both apoptosis and necrosis. This type of cell death is called necroptosis and occurs by activating the extrinsic pathway when apoptosis is inhibited. Similar to necrosis, it induces cell and organelle swelling with rupture of the plasma membrane [29,30,31,32]. This will cause an intense inflammatory reaction that exacerbates the damage in the infarcted myocardium [17,33]. Autophagy seems to play a significant yet controversial role in the pathogenesis of ventricular remodeling after MI. Under stress conditions, autophagy acts as an adaptive mechanism, as it removes the damaged organelles and defective proteins by sequestrating the altered cell content and delivering it to lysosomes. This way, the cell content is degraded into amino acids and fatty acids that will be used for energy production and protein synthesis [34,35,36]. During MI, the magnitude of the process depends on the severity and duration of the ischemic event. Moderate hypoxia seems to induce modest levels of autophagy that will protect the cardiomyocytes from cell death, as autophagy prevents apoptosis [37]. During severe ischemic conditions, inhibition of autophagy activates cell death and leads to poor myocardial performance [38,39]. 

### 2.2. Inflammation

Following acute MI, the inflammatory response plays a critical role in the healing process of the infarcted myocardium, as well as in the progression of adverse remodeling. Myocardial necrosis triggers both systemic and local inflammatory responses to clear the myocardial tissue from dead cells and matrix debris. The releasing of endogenous danger molecules called damage-associated molecular patterns (DAMPs) will activate the complement cascade and toll-like receptor (TLRs)/ interleukin-1 (IL-1) signaling, which will stimulate the synthesis of pro-inflammatory cytokines such as TNF-α, interleukin-1β (IL-1β), and interleukin-6 (IL-6) [40]. DAMPs will also activate pyrin domain- containing protein 3 (NLRP3) inflammasome which plays a major role in the emergence of sterile inflammation, as it triggers the production of IL-1β. Once activated in cardiomyocytes, NLRP3 will activate caspase 1, which will mediate pyroptosis, a form of cell death that combines characteristics of both apoptosis and necrosis [41,42]. 

Studies have shown that levels of pro-inflammatory cytokines are correlated with the development of LV remodeling after MI. In post-infarct mice, IL-1β secretion was stimulated after the onset of the ischemic event [40]. Also, IL-1β levels were correlated with impaired LV function 1 year post-ST segment elevation myocardial infarction (STEMI) [43]. The production of pro-inflammatory cytokines will further cause the secretion of chemokines and adhesion molecules that are vital for the recruitment of inflammatory cells in the infarcted area. The first cells to arrive within hours following MI are neutrophils, which phagocytize the necrotic cardiomyocytes and degrade the extracellular matrix (ECM) [44,45]. In addition, by secreting chemotactic factors, neutrophils regulate the inflammatory response and modulate the recruitment of monocytes that will differentiate into inflammatory macrophages [46,47]. More specifically, the healing process is coordinated by two macrophage phenotypes. M1-phenotype is responsible for the pro-inflammatory reaction as M1-macrophages not only release pro-inflammatory cytokines like TNF-α and IL-1β, but also phagocytize the necrotic debris through a different apoptotic process called efferocytosis [48,49,50]. This particular process promotes the activation of signaling pathways which will polarize macrophages to an M2 anti-inflammatory phenotype responsible for the secretion of anti-inflammatory cytokines and growth factors and for marking the beginning of the repair phase [49,51]. However, this process does not always lead to normal healing of the infarcted tissue, as M1/M2 macrophages’ imbalance can trigger an excessive and prolonged inflammatory reaction that will increase tissue destruction and promote progression to adverse remodeling [43,52]. 

Adaptive immunity influences the remodeling process following acute MI as well, with the role of T cells being the most extensively evaluated. After interacting with the antigen-presenting cells, T cells will differentiate into either cytotoxic T cells (CD8+ cells) or T-helper (Th) cells (CD4+ cells) and will infiltrate the injured myocardium [53,54,55]. Th cells arrive in the infarcted area and promote myocardial repair in the early stages of MI. In line with this, preclinical research demonstrated that CD4+ deficient mice presented abnormal collagen deposition and myocardial scar development, which further led to LV dilatation [56,57]. Also, the imbalance between different subsets of Th cells is responsible for the transition to pathological remodeling. Th1 and Th17 subsets produce inflammatory cytokines which can result in adverse myocardial remodeling in prolonged inflammatory states, while Th2 cells present an anti-inflammatory profile. Another subset of CD4+ lymphocytes are the regulatory T (Treg) cells, which limit the inflammatory response by directly suppressing activated effector T cells or through the inhibition of antigen-presenting cells [55,58,59,60]. Their protective effect on the ischemic myocardium was suggested by experimental evidence showing that Treg depletion was associated with a pro-inflammatory M1-phenotype, increased MI size, greater LV dilatation, and adverse remodeling, while Treg cells’ activation enhanced M2 macrophage polarization, prevented excessive ECM degradation, and attenuated myocardial remodeling [55,57]. Nevertheless, in ischemic HF, the immunoregulatory function is altered, as Treg cells adopt Th1-like proinflammatory features which will detrimentally affect the remodeling process. These findings suggest that Treg cell function varies with the inflammatory stage after the ischemic injury. However, Treg cells might not transition to the pro-inflammatory phenotype in HF induced by pressure overload, suggesting that HF etiology might also affect the characteristics of Treg cells [59,60,61]. The role of cytotoxic T lymphocytes in ischemic ventricular remodeling is not completely elucidated. Following acute ischemia, these cells could exert cytotoxic activity against the normal cardiomyocytes, possibly contributing to MI expansion, and adverse remodeling, but further research is needed in order to evaluate this concept. In contrast, subsets of CD8+ T cells expressing angiotensin type 2 receptors (AT2R) ameliorate the remodeling process by downregulating the expression of pro-inflammatory molecules [58,62,63]. The contribution of B lymphocytes to the evolution of myocardial ischemic injury is poorly understood to date. Their role might be related to the production of cardiac-specific autoantibodies and modulation of the monocyte infiltration in the ischemic myocardium, which could augment the local inflammation, thus contributing to MI expansion and ventricular adverse remodeling [58].

### 2.3. Oxidative Stress 

Oxidative stress makes a major contribution to the remodeling process of the injured myocardium in ischemic disease. Normally, cells have an antioxidant defense mechanism. When the balance between oxidants and antioxidants is altered, the levels of reactive oxygen species (ROS) increase, and radicals such as superoxide anion are produced [20]. The mechanism responsible for the production of superoxide anions during ischemia involves the alteration of the mitochondrial electron transport chain which leads to the subsequent formation of additional ROS such as hydrogen peroxide and hydroxyl radical. Moreover, excessive ROS production is facilitated by enzymes like nicotinamide adenine dinucleotide phosphate (NADPH) oxidase, xanthine oxidase, and nitric oxide synthase (NOS) [64]. For instance, phagocytosis stimulates NADPH oxidase which will increase ROS release in addition to the already impaired redox status [65]. Also, when NOS becomes uncoupled under pathological conditions, its structure becomes unstable and promotes the formation of superoxide anions. 

The effects of ROS on the injured myocardium consist of the oxidation of nucleic acids, lipids, and proteins and alterations of cell membrane properties, leading to cellular dysfunction and death. Furthermore, oxidative stress stimulates matrix metalloproteinases (MMPs) expression and modulates collagen synthesis, leading to fibrosis [66]. Despite the benefic effects of reperfusion, the high consumption of antioxidants during the ischemic phase along with the abrupt exposure of the hypoxic cells to oxygen generate the highest levels of ROS, which promotes myocardial necrosis in the next days after reperfusion, making oxidative stress a central element in the pathogenesis of ischemia/reperfusion (I/R) injury [67,68,69]. 

### 2.4. Fibrosis 

Fibroblasts are generally known for producing and organizing the ECM. Fibroblasts act as regulators of myocardial remodeling after an ischemic event. Under pathological conditions, fibroblasts are activated into myofibroblasts with the capacity to remodel the ECM through activated integrins and cadherin receptor proteins. During the early phase of MI, cytokine secretion will contribute to the accumulation of fibroblasts in the injured area [70,71]. However, at this stage, fibroblasts are not able to differentiate into myofibroblasts due to the high secretion of IL-1, which delays this process until the infarcted area is cleared of necrotic debris. Later, in the fibrotic phase, the inflammatory cells secrete cytokines and growth factors that will initiate fibroblast differentiation into myofibroblasts which are typically absent in the healthy myocardium [72,73]. By increasing the production of anti-inflammatory and profibrotic factors such as transforming growth factor-β (TGFβ) and interleukin-10 (IL-10), myofibroblasts are stimulated to produce structural ECM proteins, especially collagen types I and III, which are fundamental for replacing the necrotic areas with the fibrotic scar and, therefore, for preventing ventricular wall rupture [74,75]. Although the process of scar formation is essential for preserving the structural integrity of the LV, the imbalance between ECM production and degradation can result in adverse myocardial remodeling, as excessive ECM production by myofibroblasts can cause impaired LV contractility and increased myocardial stiffness leading to myocardial dysfunction and, ultimately, to HF [76,77]. 

### 2.5. Neurohormonal Systems 

Neurohormonal activity serves as a key regulatory mechanism in the progression of cardiac remodeling. The activation of the sympathetic nervous system (SNS) and renin-angiotensin-aldosterone (RAAS) system occurs initially as a compensatory mechanism in the acute decompensated state to become later a maladaptive process. While in the acute phase the compensatory action of SNS aims to maintain an adequate stroke volume, prolonged sympathetic activity can sustain the ventricular remodeling by enhancing the apoptotic pathways, promoting the release of proinflammatory cytokines, and impairing the myocardial contractility. Also, chronic stimulation induces myocardial alterations by promoting RAAS activation [16,78]. Renin and angiotensin-converting enzyme (ACE) are produced in both infarcted and noninfarcted myocardial tissue. The generation of angiotensin II will activate the angiotensin type 1 receptors (AT1R), which stimulate fibroblast proliferation and ECM protein synthesis, leading to fibrosis. In addition, aldosterone promotes fibrosis by directly stimulating fibroblast proliferation and enhancing collagen production [79,80,81]. All of these processes will alter the left ventricular configuration, from an initial elliptical to a spherical shape, and dilatation with increased end-systolic and end-diastolic volumes and reduced cardiac output [17]. 

## 3. Novel Therapeutic Strategies in IHD with Reduced EF 

**Figure 2:**
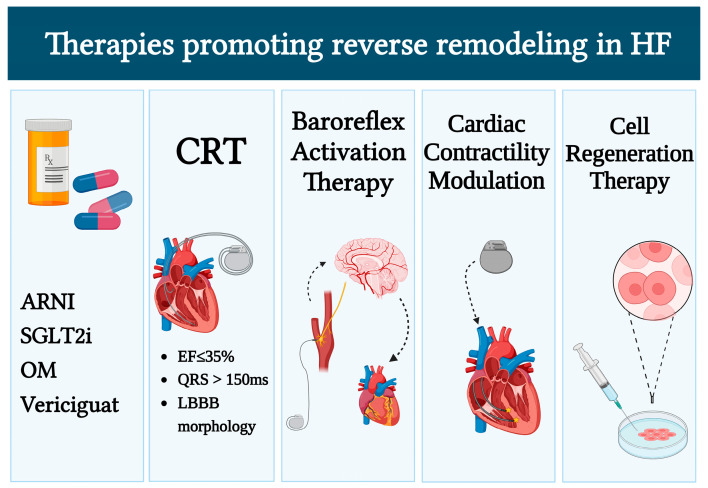
Novel HFrEF strategies with a potential role in IHD reverse remodeling. ARNI, angiotensin receptor neprilysin inhibitor; CRT, cardiac resynchronization therapy; EF, ejection fraction; LBBB, left bundle branch block; HF, heart failure; OM, omecamtiv mecarbil; SGLT2i, sodium-glucose co-transporter 2 inhibitors.

**Figure 3:**
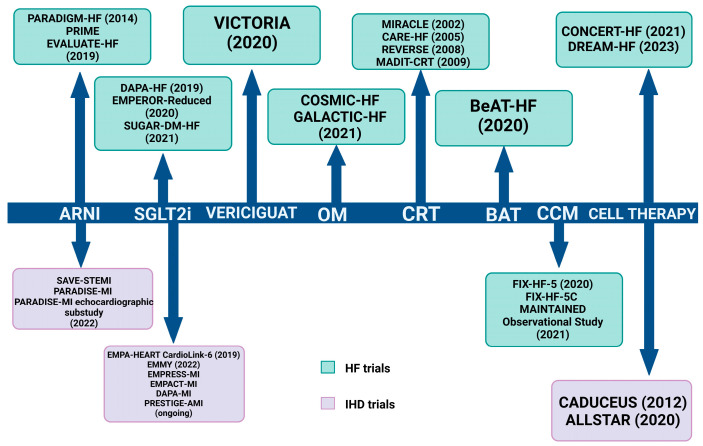
Major trials of novel HFrEF strategies and trials that evaluate these therapies in IHD. ARNI, angiotensin receptor neprilysin inhibitor; SGLT2i, sodium-glucose co-transporter 2 inhibitor; OM, omecamtiv mecarbil; CRT, cardiac resynchronization therapy; BAT, baroreflex activation therapy; CCM, cardiac contractility modulation.

Detrimental ventricular remodeling following ischemic injury is a key contributor to HFrEF progression. Traditional HFrEF therapies, which include beta-blockers, ACE inhibitors (ACEI), angiotensin II receptor blockers (ARB), and mineralocorticoid receptor antagonists (MRA), have extensive data from clinical trials to support their beneficial effect in patients with IHD and HFrEF, translated into improvements in survival and ventricular remodeling and function [82,83]. More recently, the treatment of HFrEF has been innovated by the introduction of novel pharmacological therapies and device strategies which have been demonstrated to ameliorate the prognosis of HFrEF patients (Figure 2). In the following part of this manuscript, we will discuss current knowledge on the role of these therapies in HFrEF and IHD, as well as their potential role to promote reverse remodeling [84]. The major trials that evaluate the efficacy of these therapies are summarized in Figure 3. 

### 3.1. Pharmacologic Therapies

#### 3.1.1. Angiotensin Receptor Neprilysin Inhibitor

ARNI is a dual regulator that inhibits the RAAS as well as the breakdown of physiologically active natriuretic peptides and several other vasoactive substances, including bradykinin. The rise in circulating natriuretic peptides caused by neprilysin inhibition leads to vasodilatation, natriuresis, and lowering of intracardiac pressures, while blocking the angiotensin receptors type 1 exerts antiproliferative, antihypertrophic, and antifibrotic effects, all of these mechanisms promoting favorable ventricular remodeling [85,86,87]. The beneficial effect of the first drug in this therapeutic class, sacubitril/valsartan, has been proven by the PARADIGM-HF trial. This study included symptomatic patients with New York Heart Association (NYHA) class II, III, or IV HF and an LV EF of 40% or less, that were randomized to receive either sacubitril/valsartan or enalapril in addition to standard therapy. IHD was the etiology of HF in 59.9% of the patients from the sacubitril/valsartan group and in 60.1% of the patients from the enalapril group. The results of the trial showed that sacubitril/valsartan was superior to enalapril in decreasing the risk of death from cardiovascular causes or hospitalization for HF, which was reduced by 20% and 21%, respectively. The beneficial effect of sacubitril/valsartan was independent of the etiology of HF, whether ischemic or non-ischemic [88,89]. The compelling results of this trial led to the European Society of Cardiology (ESC) recommendation to replace ACEI or ARB therapy with sacubitril/valsartan in patients with HFrEF who remain symptomatic under optimal therapy [84].

Several small studies demonstrated the positive effects of ARNI on cardiac remodeling, as suggested by improvements in LV EF and LV end-systolic and end-diastolic volumes and diameters after therapy with sacubitril/valsartan [90,91]. Subsequently, the randomized PRIME trial sought to evaluate whether sacubitril/valsartan is superior to valsartan alone in reducing functional mitral regurgitation (MR) in symptomatic patients with HF and an EF between 25–50%. Patients with ischemic MR were 36.7% in the sacubitril/valsartan group and 34.5% in the valsartan group. The results showed that sacubitril/valsartan therapy was associated with a significantly greater decrease in the effective regurgitant orifice area and regurgitant volume compared to the control group. Although at follow-up the LV end-systolic volume (LVESV) and LV end-diastolic volume (LVEDV) were significantly smaller in the sacubitril/valsartan group, secondary analysis did not identify significant differences in the changes of LVESV and LVEDV between groups, except for the decrease in LVEDV index (LVEDVI) which tended to be greater in the sacubitril/valsartan group. By decreasing ischemic MR severity, sacubitril/valsartan could promote reverse ventricular remodeling in IHD and improve HF symptoms [92]. The EVALUATE HF trial was another randomized study centered on evaluating cardiac remodeling in patients with HF and an EF of less than 40% that were randomized to receive sacubitril/valsartan or enalapril for 12 weeks. IHD was the etiology of HF in 59% of patients from the ARNI group and in 63% of patients in the control group. Although no significant between-group difference in change from baseline was seen in LV EF (34% to 36% with sacubitril-valsartan vs. 33 to 35% with enalapril), other parameters of cardiac remodeling had a significantly greater reduction with sacubitril/valsartan compared to enalapril, such as the left atrial volume (from 30.4 mL/m^2^ to 28.2 mL/m^2^ vs. from 29.8 mL/m^2^ to 30.5 mL/m^2^), the LVEDVI (from 75.1 mL/m^2^ to 70.3 mL/m^2^ vs. from 79.1 mL/m^2^ to 75.6 mL/m^2^), and the LVESV index (LVESVI) (from 50.8 mL/m^2^ to 46.3 mL/m^2^ vs. from 54.1 to 50.6 mL/m^2^). These results suggest that sacubitril/valsartan is superior to ACEI in promoting LV reverse remodeling, regardless of the etiology of HF, being a valuable therapy to consider for counteracting the adverse remodeling process associated with chronic IHD [93].

Considering the tremendous benefits of ARNI in the management of HFrEF, including the ischemic etiology, several trials sought to investigate the potential role of sacubitril/valsartan in acute MI. The SAVE-STEMI trial evaluated the safety and efficacy of ARNI compared to ramipril in 200 patients with STEMI after undergoing primary percutaneous coronary intervention. The primary endpoint of major adverse cardiac events (MACE) representing a composite of cardiac death, MI, and HF hospitalizations was similar between groups at 30 days, while at 6 months, it was significantly reduced with sacubitril/valsartan, mainly driven by the reduction in HF hospitalizations. In addition, at 6 months, sacubitril/valsartan was associated with a significantly greater improvement in LV EF, LV end-diastolic diameter (LVEDD), and LV end-systolic diameter (LVESD) [94]. 

A subsequent trial that further evaluated the benefits of ARNI in MI was the PARADISE-MI trial, which included 5661 patients with acute MI with reduced LV EF of less than 40%, pulmonary congestion associated with the index acute MI, or both. The patients were randomly assigned to receive sacubitril/valsartan or ramipril. The primary outcome was cardiovascular death or an HF episode (requiring outpatient management or hospitalization). During a mean observation period of 22 months, a primary-outcome event occurred in 11.9% of patients in the sacubitril-valsartan group and in 13.2% of patients in the ramipril group (hazard ratio, 0.90; 95% confidence interval [CI], 0.78 to 1.04; *p* = 0.17), leading to the conclusion that sacubitril-valsartan was not superior to ramipril in reducing the incidence of cardiovascular death or incident HF in patients with acute MI [95]. However, the PARADISE-MI echocardiographic substudy, which evaluated the effect of sacubitril/valsartan compared with ramipril on LV function and adverse remodeling, revealed several benefits of the combination therapy. Although there was no significant difference in terms of change in LV EF or left atrial volume between groups, patients from the sacubitril/valsartan group had less increase in LVEDV. In addition, they had a greater decline in LV mass index, an increase in lateral tissue Doppler velocity, and a decrease in tricuspid regurgitation peak velocity compared to patients from the ramipril group. These results demonstrated that sacubitril/valsartan is associated with less increase in LV dimensions and with improved diastolic function in patients with acute MI [96].

A recent meta-analysis which included 13 studies evaluated the efficacy of ARNI in reducing MACE and improving LV remodeling in patients with acute MI complicated with HF. The results showed that treatment with sacubitril-valsartan improved LV EF and decreased the LV remodeling parameters (LVEDD, LVESVI, and LVEDVI) to a greater proportion compared to the control group. In addition, sacubitril/ valsartan was associated with lower levels of N-terminal pro-B-type natriuretic peptide (NT-proBNP) and with a greater increase in exercise capacity. Moreover, it further reduced the incidence of adverse cardiovascular events and the rate of HF rehospitalization compared to ACEI/ARB without significantly reducing the incidence of cardiac death or MI recurrence [97]. 

#### 3.1.2. Sodium-Glucose Co-Transporter-2 Inhibitors

SGLT2 inhibitors are therapeutic agents originally designed for the treatment of type 2 diabetes (T2D) which exert their hypoglycemic effect by inhibiting glucose reabsorption in the proximal convoluted tubule, subsequently promoting glycosuria and lowering plasmatic glucose levels [98,99,100]. More recently, SGLT2 inhibitors were demonstrated to exert cardioprotective effects, although their exact mechanisms are not fully understood. Several hypotheses to explain these effects were generated, such as decreased preload and afterload due to natriuresis and reduction in blood pressure, improvement in cardiac energy metabolism and coronary endothelial function, increased cardiomyocyte autophagy and lysosomal activity, reduced oxidative stress and inflammation, and increased erythropoiesis with subsequent augmentation of the blood oxygen supply [101,102,103].

The tremendous cardiac benefits of SGLT2 inhibitors emerged for the first time from the major cardiovascular safety trials of these drugs, such as DECLARE-TIMI 58 and EMPA-REG OUTCOME, which showed that SGLT2 inhibitors therapy was associated with remarkable improvements in cardiovascular outcomes, including a reduction in mortality and HF hospitalization [104,105].

Later, the effects of dapagliflozin in HF were studied in the DAPA-HF trial, which included patients with HF with a reduced EF of 40% or less, regardless of the presence of T2D. In this trial, dapagliflozin reduced the risk of HF hospitalization and cardiovascular death with similar efficacy in patients with or without diabetes [106]. Similar results were obtained in the EMPEROR-Reduced trial, in which empagliflozin reduced the combined risk of cardiovascular death and hospitalization for HF by 25% in patients with HFrEF. Additionally, SGTL2 inhibitors appear to be more effective than vericiguat and comparable to ARNI in preventing HF hospitalization [101,106,107]. Both of these trials included more than half of the patients with an ischemic etiology of HF, and post hoc subgroup analysis showed similar outcomes in patients with or without ischemic HF [108].

Considering the categorical benefits of SGLT2 inhibitors in cardiovascular mortality and HF prognosis, several studies were designed to evaluate the impact of these drugs on cardiac function and remodeling. The SUGAR-DM-HF trial included patients with NYHA class II to IV HF, LV EF ≤ 40%, and T2D or prediabetes, that were randomly assigned to receive empagliflozin or placebo. After 36 weeks of treatment, empagliflozin significantly reduced the LVESVI and LVEDVI (assessed with cardiac magnetic resonance) compared to placebo, suggesting that SGLT2 inhibitors promote LV reverse remodeling, which might explain their benefits on cardiovascular mortality and HF hospitalizations [109]. Another study, the EMPA-HEART CardioLink-6, which included patients with T2D and coronary artery disease (CAD), demonstrated that a 6-month treatment with empagliflozin was associated with a reduction of the LV mass index, assessed by cardiac magnetic resonance [105,109]. Several hypotheses have been proposed to explain the anti-remodeling effects of SGLT2 inhibitors. Preclinical data suggest that SGLT2 inhibitors might reduce cardiac inflammation decreasing the macrophage inflammatory response, which will subsequently reduce the ECM turnover, preventing or ameliorating cardiac remodeling. In addition, SGLT2 inhibitors suppress pro-fibrotic markers, such as type 1 collagen and MMPs, and attenuate TGF-β1-induced fibroblast activation, thus reducing myocardial fibrosis [110,111].

As SGLT2 inhibitors appear to exert pleiotropic cardiovascular effects, their role in acute myocardial ischemia was evaluated in several trials. The EMMY trial included 476 patients with acute MI that were randomized to receive empagliflozin or placebo 3 days after interventional coronary revascularization. The primary outcome was represented by the change in NT-proBNP value after 26 weeks, while secondary outcomes included changes in echocardiographic parameters. The results showed a significantly greater reduction of NT-proBNP in the empagliflozin group. In addition, markers of ventricular remodeling such as LV EF, LVESV, LVEDV, and LV filling pressures improved with a greater magnitude in patients with empagliflozin therapy. These results suggest that the early initiation of the SGTL2 inhibitors in the treatment of acute MI could be beneficial for the prevention or attenuation of LV remodeling [112,113]. Other ongoing trials will provide, in the future, the necessary data to conclude the efficacity of SGLT2 inhibitors in the prevention of HF after acute MI. The EMPACT-MI trial (NCT04509674) will evaluate whether empagliflozin compared to placebo can lower the risk of HF and death in patients with acute MI and new onset LV systolic dysfunction or signs and symptoms of pulmonary congestion [114]. The DAPA-MI trial (NCT04564742) will offer information about the efficacity of dapagliflozin compared to placebo in the prevention of HF hospitalization or CV death in patients with acute MI and evidence of impaired LV systolic function. An important aspect related to these two trials is that the DAPA-MI trial will randomize only patients without a known diagnosis or evidence of T2D, while EMPACT-MI will include both diabetic and non-diabetic patients [114,115]. The PRESTIGE-AMI (NCT04899479) and EMPRESS MI (NCT05020704) trials will evaluate the potential role of SGLT2 inhibitors in reducing infarct size and preventing the occurrence of LV remodeling in patients with acute MI [102]. All of these studies are of great interest since myocardial remodeling after MI continues to represent a major cause of HF progression, despite timely coronary revascularization. 

#### 3.1.3. Selective Cardiac Myosin Activators

Impaired myocardial contractility is the central pathogenic process leading to HFrEF development, and therapeutic strategies which increase myocardial contraction should have a positive therapeutic impact. However, traditional inotropic drugs were not demonstrated to be beneficial for chronic administration in HFrEF, and they have been linked to an increased risk of morbidity and mortality due to increased myocardial oxygen consumption and myocyte toxicity as a consequence of intracellular calcium overload [116]. Omecamtiv mecarbil is the first drug from the class of selective cardiac myosin activators, which boosts myocardial force production by modulating the function of the sarcomere without affecting the intracellular calcium accumulation. The central mechanism of omecamtiv mecarbil is to activate myosin by accelerating the rate of adenosine triphosphate (ATP) hydrolysis to adenosine diphosphate (ADP) and phosphate, subsequently increasing the number of strong actin–myosin interactions, which result in augmented myocyte contraction with increased ventricular force generation and systolic ejection time [116,117,118,119].

The COSMIC-HF study was a phase 2 clinical trial that evaluated the pharmacokinetics and impact on the ventricular remodeling of orally administered omecamtiv mecarbil in patients with HFrEF. After 20 weeks of treatment, omecamtiv mecarbil compared to placebo was associated with increased systolic ejection time and stroke volume and improvements in LVESD and LVEDD, suggesting a beneficial effect of omecamtiv mecarbil on cardiac function and LV remodeling [120,121]. In addition, patients from the omecamtiv mecarbil group presented lower plasma concentrations of NT-proBNP and decreased heart rate values, suggesting that the enhancement of systolic function can minimize myocardial wall stress and possibly sympathetic activation, which might contribute to the favorable remodeling effect of this drug. This trial included a significant proportion of patients with IHD (65%), which suggests that omecamtiv mecarbil promotes positive ventricular remodeling in patients with HFrEF with an ischemic etiology as well [120,121].

The GALACTIC-HF phase 3 trial evaluated the cardiovascular outcomes of omecamtiv mecarbil compared to placebo in patients with symptomatic HF and an EF of less than 35%. The results showed that patients who received omecamtiv mecarbil had a lower incidence of a composite of HF events or cardiovascular death compared to placebo. In addition, NT-proBNP had lower values in the omecamtiv mecarbil group, and the clinical benefit was greater among patients with LVEF ≤ 28% and systolic blood pressure ≤ 100 mm Hg. Half of the included patients had ischemic HF. There were no considerable differences between groups regarding the occurrence of cardiac ischemic events and ventricular arrhythmias [119,122,123]. Despite the aforementioned positive effects of omecamtiv mecarbil in HFrEF patients, the recently published results of the METEORIC-HF trial showed that omecamtiv mecarbil did not significantly improve exercise capacity over 20 weeks compared with placebo [124].

There is no data in the literature specifically analyzing the role of omecamtiv mecarbil in the management of acute or chronic IHD. Scarce evidence coming from experimental studies suggests that omecamtiv mecarbil could have cardioprotective properties against I/R injury [125]. 

#### 3.1.4. Soluble Guanylate Cyclase Stimulators

Soluble guanylate cyclase (sGC) stimulators emerge as a valuable treatment option in patients with impaired LV systolic function. HFrEF is associated with decreased cyclic guanosine monophosphate (cGMP) production, which promotes myocardial dysfunction and abnormal vasomotor regulation. cGMP deficiency is caused by altered nitric oxide (NO)-sGC-cGMP pathway signaling, due to decreased levels of NO in the context of the endothelial dysfunction state, which characterizes HFrEF [126]. By directly stimulating sGC independent of NO, these drugs increase cGMP production and subsequently improve myocardial and vascular function [127,128,129].

Vericiguat is an oral sGC stimulator approved for the treatment of HFrEF, following the results of the VICTORIA trial. This phase III trial included patients with EF < 45% and a history of recent episodes of HF decompensation and showed that vericiguat added to standard therapy is associated with a lower incidence of the primary composite outcome of cardiovascular death or HF hospitalization compared to placebo added to standard therapy [126]. A post hoc study showed that IHD defined as previous MI, surgical, or percutaneous coronary revascularization, was the etiology of HFrEF in more than half of the included patients. In addition, it showed that patients with coronary artery disease benefited as well from vericiguat therapy, as they had a lower primary outcome of cardiovascular death and HF hospitalization compared to patients with CAD from the placebo group [130].

The potential effect of vericiguat on ventricular remodeling was evaluated in the VICTORIA echocardiographic substudy, in which echocardiographic evaluation was performed at baseline and after 8 months of therapy in the two groups of patients with vericiguat and placebo therapy, respectively. The results failed to demonstrate a superior effect of vericiguat compared to placebo on ventricular function and dimensions. Although treatment with vericiguat significantly improved LV EF and LVESVI after 8 months, it did not show an additional significant effect on LV EF or LVESVI compared to placebo [131]. However, when interpreting these results, one should take into consideration the particularities of the patients included in the VICTORIA trial, as they were older, less stable, and had higher NT-proBNP levels compared to the patients included in the other HF trials such as PARADIGM-HF and DAPA-HF, which demonstrated positive effects on the ventricular remodeling [131]. In addition, only 15% of patients enrolled in the VICTORIA trial were simultaneously receiving treatment with sacubitril/valsartan, only 60% were receiving guideline-based triple medical therapy, and no data was provided regarding the administration of SGLT2 inhibitors. Whether vericiguat would have a more pronounced positive effect on ventricular remodeling if added to optimal HF therapy including sacubitril/valsartan and SGLT2 inhibitors, and in a more specific group, with less unstable patients, are questions that merit to be addressed in future studies [127,128,129].

Currently, there are no trials evaluating the effects of sGC stimulators on cardiac remodeling and HF development in patients with acute MI. However, data coming from experimental studies suggest a potential beneficial effect of sGC stimulators on ventricular remodeling after acute MI. In this regard, a study showed that riociguat administration in mice after 30 min of occlusion of the left anterior descending artery (LAD) followed by reperfusion therapy, resulted in a reduction of the infarct size and prevented the further development of HF. The beneficial effect was persistent, as the LV systolic function was preserved at 28 days after the ischemic event [132,133]. Similarly, another study evaluated the effects on the cardiac remodeling of ataciguat compared to either placebo, ramipril, or a combination of both in rats, with the initiation of therapy 10 days after acute MI. After 9 weeks, ataciguat administration was associated with lower LVESV and LVEDV and improved systolic and diastolic function compared to placebo or ramipril. In addition, these effects were potentiated when ataciguat was added to the ACEI. At a cellular level, ataciguat was demonstrated to reduce mitochondrial oxidative stress production, cardiomyocyte hypertrophy, interstitial collagen accumulation, and fibrosis development [134,135]. These results suggest a potential protective role of sGC stimulators against cardiac fibrosis and maladaptive ventricular remodeling in chronic IHD, which could be further explored in future research. 

### 3.2. Device Therapy in HF

#### 3.2.1. Cardiac Resynchronization Therapy 

Cardiac resynchronization therapy (CRT) is a valuable therapeutic resource in the management of patients with HFrEF and wide QRS complex that remain symptomatic despite guideline-directed medical therapy (GDMT). Biventricular pacing improves intraventricular and interventricular synchrony and increases diastolic filling time, which overall translates into improved cardiac performance and amelioration of symptoms [136]. Multiple randomized clinical trials demonstrated the beneficial effects of CRT. Response to therapy was defined by clinical measures (improvements in NYHA class, quality of life, or 6 min walk test), LV reverse remodeling parameters (improvements in LV EF, LVESV, LVEDV, and mitral regurgitation), or by outcome measures, including reductions in HF hospitalizations, and mortality [136]. Response rates have varied across studies, depending on the criteria utilized to define response to therapy. As such, response rates were higher when clinical measures were used, while remodeling and outcomes indicators were associated with lower rates of response. In addition, clinical response was not always accompanied by improved ventricular remodeling or survival. Among 20 to 40% of patients with CRT remain unresponsive, which has been attributed to factors such as IHD with extensive myocardial scarring, atrial fibrillation or atrial conduction delay, severe ventricular dilatation, or mitral regurgitation [137,138].

Patients with IHD represented over 50% of the patients included in every trial evaluating CRT, except for CARE-HF, in which IHD was the etiology of HF in 36% of the patients [139]. The trials which particularly addressed the effect of CRT on ventricular remodeling will be presented below.

The CARE-HF trial included patients with symptomatic HF NYHA class III or IV, prolonged QRS, and a reduced LV EF < 35%, which were randomized to receive CRT plus optimal medical therapy, and standard HF therapy alone. The primary endpoint was a composite of death from any cause or an unplanned hospitalization for a major cardiovascular event. After a mean period of follow-up of 29.4 months, the primary endpoint was significantly lower in the CRT group compared to the group with medical therapy alone (39% vs. 55%). In addition, CRT was associated with clinical response, attributed to improved symptoms and quality of life, and with LV reverse remodeling, measured as a reduction in LVESVI, and in the area of the mitral regurgitant jet, or an increase in the LV EF [140].

The MIRACLE trial was among the first to show the clinical benefits of CRT. It included patients with NYHA class III or IV, LV EF of 35% or less, and a QRS duration of 130 msec or more, that were randomized in a CRT group and a control group with the maintenance of conventional HF therapy in both groups. Patients with IHD represented 58% of the total number of included patients [139,141]. The primary endpoints were the NYHA functional class, quality of life, and the distance walked in six minutes, which were significantly improved in the CRT group compared to the control group. The extent of the effect of CRT on the primary endpoints was not influenced by the etiology of HF, whether ischemic or nonischemic. In addition, CRT enhanced cardiac performance and promoted ventricular remodeling, evaluated as an increase in the LV EF and a reduction in the end-diastolic LV dimension and the area of the mitral regurgitant jet [141].

MADIT-CRT was another trial that included patients with ischemic HF (NYHA class I or II) and nonischemic HF (NYHA class II only), an LV EF of 30% or less, and a QRS duration of 130 msec or more. Patients with IHD represented 55% of the total included patients. They were randomized to receive CRT plus an implantable cardioverter-defibrillator (ICD) or an ICD alone. The primary endpoint was death from any cause or a nonfatal HF event. The results showed that CRT-ICD was superior to ICD alone, as it reduced the risk of death or HF events by 34%. In addition, CRT was demonstrated to favor LV reverse remodeling, as the LV volumes were reduced and the EF was increased to a greater amount in patients with a CRT-ICD compared with those with an ICD only [142]. However, when assessing the response to CRT by the etiology of HF, it appeared that patients with non-IHD had a greater clinical benefit compared to patients with IHD, with a reduction in the risk of HF or death of 44% and 34%, respectively. Moreover, the echocardiographic response to CRT was of smaller magnitude in patients with IHD compared to patients with non-IHD, with reductions in LVESV of 29 ± 14% vs. 37 ± 16%, and in LVEDV of 18 ± 10% vs. 24 ± 12%, respectively [143].

The REVERSE trial included patients with mildly symptomatic HF (NYHA class I or II) and a LV EF ≤ 40% who received a CRT device and were randomly assigned to active CRT (CRT-ON) or control (CRT-OFF) for 12 months. The results showed that patients from the CRT-ON group had a lower risk of HF hospitalization, as the worsened HF clinical response was lower in this group compared to the CRT-OFF group. In addition, patients who had active CRT had greater improvement in the LVESVI and other markers of ventricular remodeling. Notably, the improvement in LVESVI in CRT-ON patients was 3 times greater in the nonischemic group than in patients with IHD [144].

Sub-analysis of the trials presented above showed that indeed, CRT is associated with a greater LV reverse remodeling in nonischemic HF, compared to the ischemic etiology. Integrating data from the MADIT-CRT trial, seven factors were identified to predict echocardiographic response to CRT with defibrillation (CRT-D): female sex, nonischemic origin, left bundle-branch block (LBBB), QRS ≥ 150 msec, prior hospitalization for HF, LVEDVI ≥ 125 mL/m^2^ and left atrial volume < 40 mL/m^2^ [145]. However, new strategies are emerging to improve the ventricular remodeling response to CRT in IHD. In line with this, using two-dimensional speckle tracking imaging for LV lead position to the site of the latest activation has been associated with significantly greater reverse remodeling compared to the CRT procedure without echocardiographic guidance [146]. Moreover, sequential biventricular pacing with optimization of interventricular pacing interval (V-V) was demonstrated to improve ventricular systolic performance with a greater increase in LV EF compared to conventional simultaneous stimulation, and to a greater extent in patients with IHD compared to patients with non-IHD [139,147].

Considering the compelling data from clinical trials, current European and American guidelines recommend CRT with a class I indication in patients with symptomatic HF despite optimal medical therapy, with reduced ejection fraction (LV EF < 35%), and a QRS with LBBB morphology and a duration ≥ 150 msec [83,148]. 

#### 3.2.2. Baroreflex Activation Therapy

Sympathetic overactivation is a major contributor to the progression of HF and ventricular maladaptive remodeling. Baroreflex activation therapy (BAT) emerges as a novel therapeutic approach in HFrEF, consisting in carotid baroreceptor stimulation, which subsequently decreases the sympathetic hyperreactivity and augments the parasympathetic tone, rebalancing the cardiac autonomic modulation [149,150]. BAT with Barostim Neo, an implantable system composed of a pulse generator and a carotid sinus lead that delivers electrical impulses to the carotid artery baroreceptors, has recently been evaluated in the phase III BeAT-HF trial. This study included patients with symptomatic HF NYHA class II or III, a LV EF ≤ 35%, and no Class I indication for CRT. The patients were randomized to receive either BAT plus optimal medical therapy or optimal medical therapy alone. The results showed that patients from the BAT group had significant improvements in quality of life, exercise capacity, and NT-proBNP levels, which led to the Food and Drug Administration (FDA) Barostim Neo approval in this category of patients [151].

While IHD was the etiology of HF in 66% of the included patients, there is no specific data regarding the particular response to BAT of patients with ischemic cardiomyopathy. Furthermore, the LV EF and dimensions were not endpoints in the BeAT-HF trial, therefore there is no data regarding the possible LV remodeling in the BAT group [151]. However, given the fact that BAT decreases sympathetic hyperreactivity, which is an aggravating factor of ventricular remodeling, and that it is associated with the lowering of NT-proBNP values, it would be interesting to assess in future trials whether BAT promotes or augments LV remodeling.

#### 3.2.3. Cardiac Contractility Modulation (CCM)

Cardiac contractility modulation (CCM) is a device-based treatment that involves delivering relatively high-voltage precisely timed electric pulses to the right ventricle (RV) septal wall for 30–40 milliseconds after cardiomyocyte activation, during the absolute refractory period of the action potential. CCM is provided by the implantable OPTIMIZER system, composed of a generator that delivers electrical signals through the two right ventricular leads [152]. The physiological mechanisms of CCM involve increasing cytosolic calcium, which strengthens sarcomeric contractions, increasing ventricular force production but without augmentation of myocardial oxygen consumption [153,154].

Initial research showing clinical benefit from CCM in patients with HFrEF led to the design of the largest clinical trial of CCM, the FIX-HF-5 study. This trial included patients with NYHA class III or IV HF, narrow QRS, and LV EF ≤ 35%, that were randomized to optimal medical therapy (OMT) plus CCM versus OMT alone. At 6 months, the primary endpoint of ventilatory anaerobic threshold (VAT) did not improve, while peak oxygen uptake (VO2) and quality of life scores significantly improved with CCM. Also, a sub-analysis identified a subgroup of patients with baseline EF ≥ 25% that derived a greater benefit from CCM [155]. This finding was further evaluated in the subsequent randomized FIX-HF-5C trial, which enrolled symptomatic NYHA class III or IV HF patients, QRS duration < 130 ms, and LV EF of 25–45%. The patients from the arm with CCM plus OMT had significant improvements in NYHA class, quality of life scores, and 6 min walk distance, compared to those with OMT alone [141]. As a result of these findings, CCM has been associated with augmentation of LV contractile performance, and it is FDA-approved for patients with NYHA class III with LV EF between 25% and 45% who are not candidates for CRT [152,153].

While these studies included a significant proportion of patients with ischemic etiology of HF, there are no available data from these trials regarding the particular response to CCM in this subgroup of patients. Recently, the MAINTAINED observational retrospective study compared the long-term effects of CCM therapy in patients with IHD versus non-IHD. After 5 years of CCM, the entire cohort had improvements in LV EF and LVEDD, but non-IHD patients showed a significantly greater augmentation of LV EF and RV systolic function, although these parameters were similarly reduced at baseline in both groups. This finding might suggest that patients with non-IHD manifest a greater functional improvement in response to CCM therapy than IHD patients [156]. The results of another observational study which included symptomatic patients with an LV EF < 35% despite OMT showed that CCM was associated with a similar LV reverse remodeling to CRT for patients with a mildly prolonged QRS, while the similarity was less strong when compared to CRT for patients with a very wide QRS [157]. Further randomized studies are needed to evaluate whether patients with IHD have a similar response to CCM as those with non-IHD, and to what extent. 

### 3.3. Cell Therapy

Despite significant progress in the pharmaceutical and device therapy of HF and the strategies of coronary revascularization in acute or chronic myocardial ischemia, a substantial proportion of patients still develop adverse ventricular remodeling, which ultimately leads to ischemic HF. As the therapeutic resources for advanced HF are limited to ventricular assist devices and heart transplantation, the finding of new therapeutic strategies for these patients is of major importance. Stem cell regeneration medicine represents a novel potential HF therapy, currently still under research, that aims to promote myocardial regeneration and repair [158]. Several mechanisms have been proposed to explain the beneficial effect of stem cell therapy in HF. While initially it was hypothesized that transplanted stem cells could promote myocardial tissue regeneration, subsequent studies demonstrated that inoculated cells do not engraft in the myocardium and do not differentiate in cardiomyocytes [159]. A considerable body of evidence later suggested that the clinical benefits of transplanted cells do not involve myogenesis but are rather related to their endocrine and paracrine effects, which activate pathways that further lead to a reduction in inflammation, attenuation of cardiac fibrosis, and promotion of cardiomyocyte survival and angiogenesis, all of these mechanisms acting in concert to improve the cardiac function [160]. 

Clinical trials utilized different types of stem cell populations to evaluate their regenerative capacity. First-generation stem cells include bone marrow-derived mononuclear cells (BM-MNCs), which were the most extensively used in clinical trials, due to their relatively easy procedure of isolation and the associated low costs. BM-MNCs include hematopoietic stem cells, endothelial progenitor cells, and a small fraction of mesenchymal stem cells (MSCs) [161]. MSCs are another type of first-generation stem cells and they are found in various tissues such as the bone marrow, the adipose tissue, and the umbilical cord matrix and blood. MSCs exert powerful paracrine actions, as they produce a large variety of cardioprotective factors which counteract inflammation, and apoptosis and stimulate angiogenesis, representing a preferred type of stem cells in clinical trials [160,161]. Research subsequently shifted towards second-generation cell therapy, which includes c-kit-positive cardiac stem cells (CSCs), cardiac progenitor cells (CPCs), and cardiosphere-derived cells (CDCs), which are isolated and expanded from cardiac tissue obtained by endomyocardial biopsy and are thought to possess increased regeneration capacity by the stimulation of endogenous cardiomyocytes or paracrine signaling [160,161,162].

#### 3.3.1. Cell Therapy in Acute Myocardial Infarction

As research on animal models suggested that cell therapy could limit infarct expansion and promote cardiac recovery in acute ischemia, several trials evaluated the potential role of cell therapy in acute MI. The most frequently explored stem cells in acute MI were BM-MNCs, followed by MSCs. Among the first trials in the field, the BOOST trial included 60 patients with acute ST-segment elevation myocardial infarction that had undergone interventional coronary revascularization and then were randomized to receive optimal medical treatment or intracoronary BM-MNCs. After 6 months, patients that received cell therapy had a greater improvement in LV EF compared to controls (6.7% vs. 0.7%), with ameliorated myocardial function in the periinfarct area [163]. However, the superiority of cell therapy was not maintained after 18 months, when reassessment showed nonsignificant differences in increases of LV EF compared to controls [164]. Similar results were obtained from a subsequent REPAIR-AMI trial, which included a cohort of 204 patients with acute MI, that were randomized to receive an infusion with BM-MNCs or placebo into the infarct artery, 3 to 7 days following coronary revascularization [165]. Patients that received intracoronary cell therapy had greater improvements in LV EF compared to the placebo group after 4 months, and lower rates of death, recurrence of MI, and revascularization procedures after 1 year. However, the majority of the following trials such as REGENERATE-AMI, MiHeart/AMI, REGENT, and BOOST 2, failed to demonstrate significant improvements in LV EF or in the clinical outcomes during the long-term follow-up in patients with acute MI that received intracoronary BM-MNCs compared to placebo [166,167,168,169,170]. The uncertain benefit of BM-MNCs therapy in acute MI was further illustrated by the results of a large meta-analysis including 41 trials, which demonstrated that cell therapy did not decrease the risk of all-cause mortality, cardiovascular mortality, or a composite measure of mortality, reinfarction, and re-hospitalization for heart failure at long-term follow-up. In addition, cell therapy did not significantly improve the quality of life or LV EF compared to the placebo group [171].

Subsequent studies explored the role of MSCs in acute MI, with expected better results, since these cells are a purer type of stem cellularity. Indeed, a meta-analysis that compared the effects of MSCs and BM-MNCs in acute MI and included a total of 36 trials demonstrated that MSCs are more effective in increasing LV EF [172]. Another recent meta-analysis that included 13 studies with MSCs in patients with acute MI, enrolling a total of 956 patients, showed that MSCs transplantation significantly improved LV EF by 3.67%, with a more pronounced effect if the procedure was performed within the first week, when LV EF increased by 5.74%. In addition, the efficacy of MSCs was similar, regardless of the delivery mode, whether transendocardial injection or intracoronary infusion. However, no significant change in LV volumes was observed, while a significant reduction in the infarct size was noted after excluding a trial with a high risk of bias [173].

Data evaluating the effect of stem cells isolated from cardiac tissue in patients after MI are scarce, due to the small number of clinical trials. CADUCEUS was the first trial of CDCs, a phase I trial that evaluated the safety of the intracoronary administration of autologous CDCs in the infarct-related artery in patients with MI and reduced LV EF. The results showed that CDCs infusion was safe, and it was associated with a reduction in scar size and an increase in the viable myocardium mass, suggesting that CDCs therapy could promote cardiac regeneration [173]. These results set the basis for the design of the larger ALLSTAR trial. This was a multicenter, randomized, double-blind trial that evaluated the safety and efficacy of intracoronary administration of allogeneic CDCs in patients with MI. A total of 134 patients with a history of MI 4 weeks to 12 months before enrollment were randomized in a 2:1 ratio to receive intracoronary CDCs or placebo. Included patients had a LV EF below 45% and a scar size greater than 15% of LV mass, as assessed with cardiac magnetic resonance. The results demonstrated that CDCs therapy is safe, as no primary safety endpoint event occurred, but it did not reduce scar size at 6 months compared to placebo. However, CDCs transfusion significantly reduced LVEDV, LVESV, and the value of NT-proBNP at 6 months, suggesting a potential role of CDCs in activating pathways that counteract the adverse ventricular remodeling process [174].

In summary, current evidence suggests that cell therapy delivered in patients with acute MI could exert cardioprotective effects, but further research is needed to determine to what extent these effects are translated into improved cardiac function and clinical outcomes.

#### 3.3.2. Cell Therapy in Ischemic Cardiomyopathy

In the setting of HF of both ischemic and non-ischemic etiology, cell therapy demonstrated promising results. Similar to the MI trials, a robust body of work consisting of multiple phase I and II trials mainly using MSCs, revealed potential benefits of cell therapy in HF, such as improvements in NYHA class, quality of life or functional capacity, and to lesser extent in LV EF and LV volumes [175]. 

CONCERT-HF was one of the landmark trials that provided strong evidence in this field. This trial was a rigorously conducted phase II, placebo-controlled, double-blind study that enrolled 125 patients with HF of ischemic etiology, with LF EF of 28.6 ± 6.1% and scar size 19.4 ± 5.8%, in NYHA class II or III, and randomized them in a 1:1:1:1 ratio to receive transendocardial injection of MSCs combined with CPCs, MSCs alone, CPCs alone, or placebo. After 12 months, the MACE were significantly reduced by CPCs alone compared with placebo, driven mainly by hospitalization for HF, and the quality of life was significantly improved by MSCs alone and MSCs combined with CPCs compared with placebo. No significant differences were observed in LV EF, LV volumes, scar size, and functional capacity among groups. These results suggest that CPCs or MSCs therapy has predictable beneficial effects in patients with ischemic HF, such as a reduction in hospitalization for HF and an improvement in quality of life [176]. 

DREAM-HF is the largest trial of cell therapy in HF up to the present, and its results were recently published in 2023. It was a phase III, randomized, double-blind, sham-controlled study that included 565 patients with ischemic (56% of the patients) or non-ischemic HF, randomized to receive a single transendocardial injection of MSCs or sham-control catheterization procedure. At baseline, the patients had a LV EF below 40% by 2-dimensional echocardiogram or below 35% by multigated acquisition scan. Although the primary endpoint was similar between groups, namely the time-to-recurrent events caused by decompensated HFrEF or ventricular arrhythmias, there were significant effects on other end-points. Compared to controls, MSCs therapy increased the LV EF in patients with baseline high-sensitivity C-reactive protein (hsCRP) ≥ 2 mg/L and improved the ventricular volumes independent of the baseline inflammatory status. In addition, MPCs decreased the risk of MI or stroke by 58% in the analysis population (HR: 0.42; 95% CI: 0.23–0.76) and by 75%, respectively, in patients with baseline hsCRP ≥ 2 mg/L (HR: 0.25; 95% CI: 0.09–0.66). It also reduced the risk of 3-point MACE by 28% (HR: 0.72; 95% CI: 0.51–1.03) in the analysis population and by 38% (HR: 0.62; 95% CI: 0.39–1.00), respectively, in patients with hsCRP ≥ 2 mg/L. These results are of tremendous importance in the development of cell therapy in HF, as they offer strong evidence of the capacity of cell therapy to reduce MACE in patients with HF. Moreover, as the beneficial effects were more pronounced in patients with baseline inflammation, this trial adds proof to the concept that MSCs reduce inflammation by paracrine stimulation, which is a major contributor to the progression of HF and adverse ventricular remodeling [177]. 

Summarizing the above, there is more and more evidence that cell therapy can positively impact the evolution of patients with HF of both ischemic and non-ischemic etiology by improving clinical outcomes, without necessarily modifying the LV systolic function or volumes [176]. Future trials are needed to further evaluate the impact of cell therapy on MACE in patients with HF as a primary endpoint, as positive results could pave the road to its introduction in the therapeutic arsenal for HF. 

## 4. Future Perspectives

As a substantial proportion of patients with acute or chronic ischemic events still develop adverse ventricular remodeling and HF despite coronary revascularization, further substantial efforts are still required to find the best therapeutic strategies to improve their cardiac performance and clinical outcomes. There are continuous advances in our current understanding of the pathophysiologic processes that lead to infarct expansion and progression of adverse remodeling in HF, and perhaps, the key to a better prognosis will be to specifically address each of these maladaptive mechanisms. While efficient counteracting therapy for certain deleterious processes, such as neurohormonal activation, is already well established, there are still unmet needs regarding other contributing mechanisms such as inflammation and increased oxidative stress. In addition, as the research field of cardiac regenerative therapies is continuously evolving, they might be part of HF therapeutic arsenal in the future. Apart from the first and second-generation cell therapies described above, next-generation cardiac regeneration strategies such as biomaterials, extracellular vesicles, endogenous generation of cardiomyocytes, and direct reprogramming of cardiac fibroblasts into cardiomyocytes emerge and are currently being tested in numerous preclinical trials, thus opening new perspectives in the treatment of HF. 

## 5. Conclusions

IHD represents the cause of chronic HFrEF in a significant proportion of patients, and improving their prognosis is a major therapeutic target. Novel HF pharmacotherapies such as ARNI and SGLT2 inhibitors were demonstrated to reduce cardiac mortality and HF hospitalizations and to promote cardiac reverse remodeling in HFrEF of both ischemic and non-ischemic etiology. When initiated early in acute MI, these drugs might play a role in promoting cardiac function recovery, limiting the progression to HF. Other novel HF therapies include omecamtiv mecarbil, a selective cardiac myosin activator, and vericiguat, a sGC stimulator, which demonstrated to improve cardiovascular outcomes and potentially ameliorate ventricular remodeling in HF of both ischemic and non-ischemic etiology. CRT reduces mortality rates and promotes ventricular reverse remodeling, and newer techniques regarding left ventricle lead positioning could augment the response rates in patients with ischemic HF. Other device therapies such as BAT and CCM can be therapeutic options for improving HF symptoms and exercise capacity, while their role in promoting reverse ventricular remodeling remains to be further explored in future studies. Cell therapy improves clinical outcomes in HF of both ischemic and non-ischemic etiology and might promote reverse ventricular remodeling. 

## Data Availability

No new data were created or analyzed in this study. Data sharing is not applicable to this article.
